# Activation of signaling receptors: do ligands bind to receptor monomer, dimer, or both?

**DOI:** 10.1186/2046-1682-6-7

**Published:** 2013-06-03

**Authors:** Xiaodong Pang, Huan-Xiang Zhou

**Affiliations:** 1 Department of Physics and Institute of Molecular Biophysics, Florida State University, Tallahassee, FL 32306, USA

## Abstract

A recent study by Dietz *et al.* using single-molecule fluorescence microscopy techniques demonstrates that, in the absence of the ligand InlB, the MET receptor exists as both a monomer and a dimer on the cell membrane, and addition of the ligand leads to increased MET dimerization. Under the crowded conditions of the cell membrane, dimer formation may be a common phenomenon for cell surface receptors. Ligand binding to both monomeric and dimeric receptors may provide parallel routes to receptor activation.

## Commentary

Cell surface receptors play important roles in the control of most fundamental cellular processes including cell cycle, fertilization, proliferation, cell migration, apoptosis, immune response, hematopoiesis, cancer, and atherosclerosis. A ligand binds to the extracellular domain (ECD) and activates the receptor. The signal then transmits into the intracellular domain (ICD) through the transmembrane domain, and stimulates a cascade of events inside the cell. Based on the presence or absence of catalytic domains, these receptors can be classified as receptors with enzymatic activity and receptors without it [1]. Receptor tyrosine kinases (RTKs) and cytokine receptors are two major types of such receptors, each with a single transmembrane helix. RTKs have a cytoplasmic catalytic domain harboring the tyrosine kinase activity. In contrast, cytokine receptors do not have intrinsic catalytic domains. Instead, a Janus kinase bound to the ICD of a cytokine receptor provides enzymatic activity. Ever since the discovery of the first RTK more than 30 years ago, intensive research efforts have led to important insights into the molecular mechanisms of receptor function [2,3]. A recent study by Dietz *et al.*[4] on the MET RTK has shed new light on the mechanism of receptor activation.

**Figure 1:**
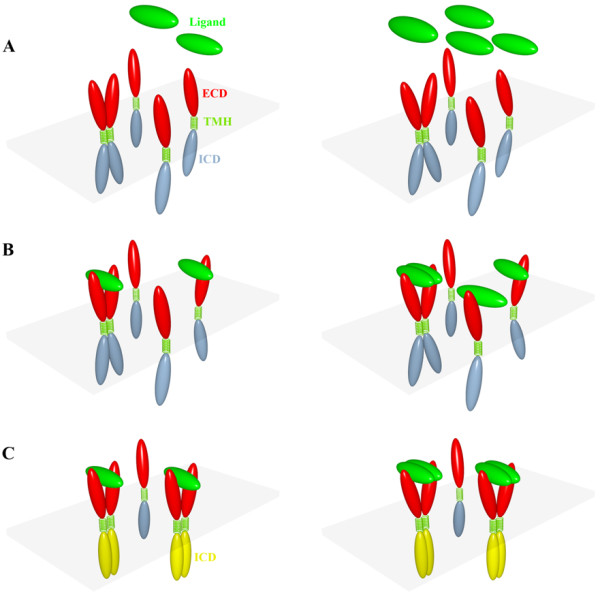
**Dual model for receptor activation.** Left panels illustrate a cytokine receptor; right panels illustrate a receptor tyrosine kinase like MET. Each receptor is composed of an extracellular domain (in red), a transmembrane helix (in lime green), and an intracellular domain (in steel blue and yellow for the inactive and active states, respectively). (**A**) Before binding the ligand (green), the receptor exists as both monomer and dimer. (**B**) The ligand binds to either monomeric or dimeric receptor. (**C**) Both binding routes lead to the same activated receptor complex.

Although sharing a common gross molecular architecture, cytokine receptors and RTKs differ in many ways. Type I cytokine receptors such as erythropoietin receptor (EPOR), growth hormone receptor (GHR), and prolactin receptor (PRLR) function as homodimers, while type II cytokine receptors are commonly heterodimers. The ECDs of type I cytokine receptors generally contain 200 to 250 amino acids forming two fibronectin-III domains linked by a hinge of a few amino acids. In contrast, the ECDs of RTKs are very diverse in terms of length and structure, typically containing a linear array of discrete folding modules such as fibronectin-III domains, immunoglobin domains, cysteine-rich domains, and epidermal-growth-factor (EGF) domains [5]. Although all the receptors are activated in an oligomeric form, different ligands employ different strategies in forming the active oligomers (Figure 1). One cytokine molecule binds to two cytokine receptors to form a complex with 1:2 stoichiometry (Figure 1*left panels*). In contrast, the RTK family is more complicated. Disulfide-linked dimeric ligands like vascular endothelial growth factor bind to their receptors to form a symmetric dimer [6,7]. Monomeric ligands like fibroblast growth factor [8] and EGF [9] form ligand:receptor complex with 2:2 stoichiometry, but dimerization is mediated only through the receptor molecules. Other monomeric ligands like bacterial ligand internalin B (InlB) [10] also bind to their receptors with 2:2 stoichiometry, but form contacts between the ligand molecules (Figure 1*right panels*). In short, the most common stoichiometry is 2:2 for RTKs and 1:2 for cytokine receptors.

Despite intensive studies, the mechanisms of receptor activation are still not completely understood. Historically two models have been proposed for receptor activation. Early studies of RTKs and cytokine receptors suggested a simple mechanism involving ligand-induced dimerization of receptors [11,12]. In the absence of ligands, the receptors were hypothesized to be maintained in a monomeric, inactive state; binding to different sites on a monomeric ligand or a ligand dimer then brought two receptor molecules together, resulting in their activation. Later studies uncovered evidence for the existence of receptors as preformed dimers, including EPOR [13], GHR [14], PRLR [15], ErbB2/Neu receptor [16], and EGF receptor [17]. These studies suggest that receptor dimerization is necessary but not sufficient for activation; activation may require conformational changes and/or relative rotation of the receptor molecules. The later model is further delineated by a computational study [18] and has gained favor in recent years.

The above two mechanistic models involve two mutually exclusive receptor states in the absence of ligand: monomer or dimer. Could the two states co-exist? The recent study by Dietz *et al.* using single-molecule fluorescence microscopy techniques clearly demonstrates that, in the absence of ligand, the MET receptor exists as both monomer and dimer on the membrane of HeLa cells, and addition of the ligand InlB leads to increased MET dimerization. This raises the question: to what form of receptor does the ligand bind? Monomer, dimer, or perhaps both? For sure, the ligand could bind to the monomer; otherwise the dimer population would not increase.

Both mechanistic models seem to have something to like in the study of Dietz *et al*. Believers in ligand-induced dimerization will take heart in the ligand-induced increase in dimer population, whereas believers in preformed dimers will be pleased by the observation of the pre-existing dimer population. One question left unanswered by Dietz *et al.* is whether InlB binds exclusively to MET monomer, or InlB could also bind to the preformed dimer. Physically it seems hard to argue why the ligand would not bind to the preformed dimer. The dimer population observed by Dietz *et al.* on the cell membrane may not be unique to MET. The cell membrane is crowded by many membrane proteins, which are expected to favor dimer formation [19]. If the large population of preformed dimer does not participate in ligand binding and is thus kept in the inactive state, then a significant fraction of the receptor would be wasted.

If the ligand binds to the receptor in both the monomeric and the dimeric form, as we suggest here, then both the proposed mechanisms can operate at the same time (Figure 1). Both monomer binding and dimer binding will lead to the same activated complex. The dimer binding route may even provide thermodynamic and kinetic advantages over the monomer binding route. Indeed, there is evidence that EGF binds dimeric EGF receptor with higher affinity than with monomeric receptor [20].

The study of Dietz *et al.* provides new insights into the mechanism of MET receptor activation. The coexistence of receptor monomer and dimer may be common for other cell surface receptors. Full understanding of receptor activation is a major challenge for the future, due to their structural and functional diversities. Much remains to be learned.

## Competing interests

The authors declare that they have no competing interests.

## Authors’ contributions

XP and HXZ wrote the paper. Both authors read and approved the final manuscript.
